# Supplementary material for: 17β-estradiol Enhances 5-Fluorouracil Anti-Cancer Activities in Colon Cancer Cell Lines
Source: Med Sci (Basel). 2022 Oct 28;10(4):62. doi: 10.3390/medsci10040062 (PMC9680382; doi:10.3390/medsci10040062)
Supplement: Supplementary file 1 [file medsci-10-00062-s001.zip › medsci-1987701-supplementary.pdf]

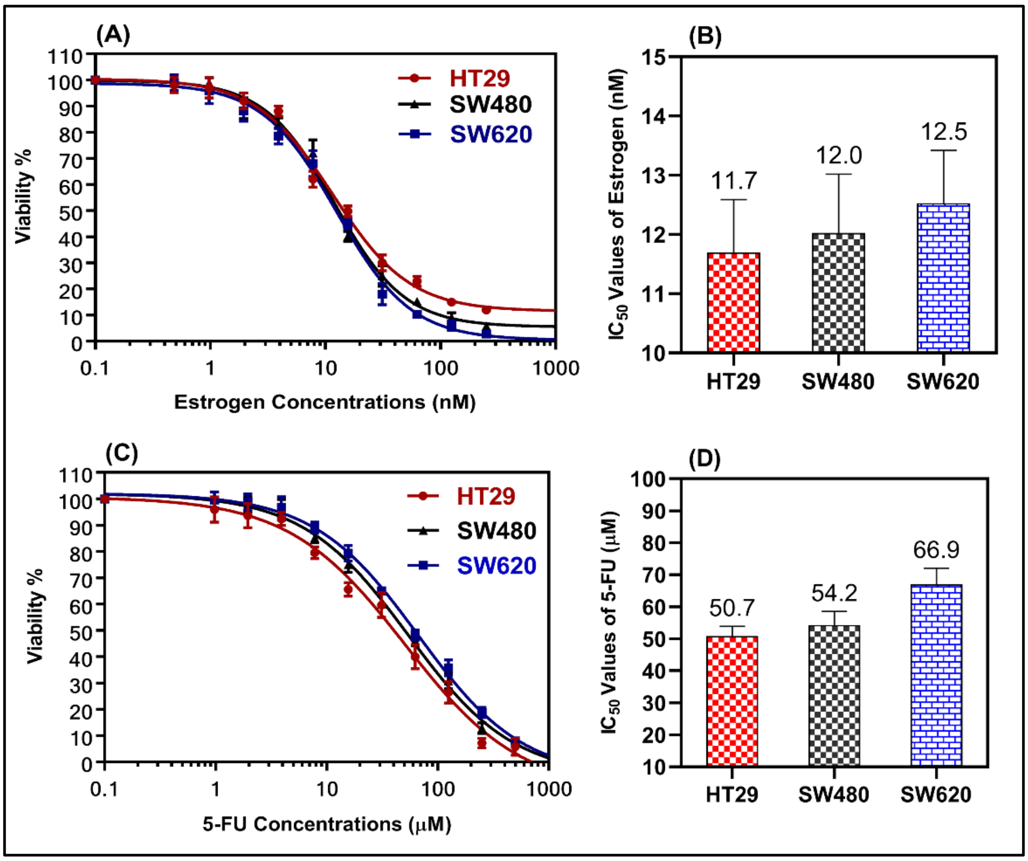

Figure S1. Colon cancer cells (HT-29, SW480, and SW620) were treated with a wide range of concentrations of the 7β-estradiol (E2) and 5-Fluorouracil (5-FU) 72 h, and the IC_{50} for each demonstrated using the MTT cell viability assay. (**A**) The MTT data showed that all the colon cancer cells have an almost similar dose response in the cell viability inhibition against the investigated concentrations of E2 monotherapy.(**B**) The IC_{50} values of the E2 were 11.7 μM for the HT-29 cells, 12 μM for the SW480 cells, and 12.5 μM for the SW620 cells. (**C**) The MTT data showed that all the colon cancer cells have an almost similar dose response in the cell viability inhibition against the investigated concentrations of 5-FU monotherapy. (**D**) The IC_{50} values of the 5-FU were 50.7 μM for the HT-29 cells, 54.2 μM for the SW480 cells, and 66.9 μM for the SW620 cells.
